# Real-Time Classification of Multivariate Olfaction Data Using Spiking Neural Networks

**DOI:** 10.3390/s19081841

**Published:** 2019-04-18

**Authors:** Anup Vanarse, Adam Osseiran, Alexander Rassau

**Affiliations:** School of Engineering, Edith Cowan University, Perth 6027, Australia; a.osseiran@ecu.edu.au (A.O.); a.rassau@ecu.edu.au (A.R.)

**Keywords:** neuromorphic olfaction, electronic nose systems, bioinspired artificial olfaction, multi-variate data classification, SNN-based classification

## Abstract

Recent studies in bioinspired artificial olfaction, especially those detailing the application of spike-based neuromorphic methods, have led to promising developments towards overcoming the limitations of traditional approaches, such as complexity in handling multivariate data, computational and power requirements, poor accuracy, and substantial delay for processing and classification of odors. Rank-order-based olfactory systems provide an interesting approach for detection of target gases by encoding multi-variate data generated by artificial olfactory systems into temporal signatures. However, the utilization of traditional pattern-matching methods and unpredictable shuffling of spikes in the rank-order impedes the performance of the system. In this paper, we present an SNN-based solution for the classification of rank-order spiking patterns to provide continuous recognition results in real-time. The SNN classifier is deployed on a neuromorphic hardware system that enables massively parallel and low-power processing on incoming rank-order patterns. Offline learning is used to store the reference rank-order patterns, and an inbuilt nearest neighbor classification logic is applied by the neurons to provide recognition results. The proposed system was evaluated using two different datasets including rank-order spiking data from previously established olfactory systems. The continuous classification that was achieved required a maximum of 12.82% of the total pattern frame to provide 96.5% accuracy in identifying corresponding target gases. Recognition results were obtained at a nominal processing latency of 16ms for each incoming spike. In addition to the clear advantages in terms of real-time operation and robustness to inconsistent rank-orders, the SNN classifier can also detect anomalies in rank-order patterns arising due to drift in sensing arrays.

## 1. Introduction

In recent years, research focusing on the development of artificial olfactory systems, also known as electronic nose systems, has gained significantly increased attention. This has been largely due to the diverse applications of these systems [1]. Whilst artificial olfactory systems emulate the biological model for sensing and processing of odor data, the biological olfactory pathway outperforms the current electronic systems in terms of accuracy and speed and can perform effectively even under rapidly changing environmental conditions [2,3]. Biological studies indicate that these attributes are achieved mainly because of the neurological architecture, which enables a rapid transformation of odor data into spikes and learning from previous responses [4]. The idea of implementing bioinspired methods for artificial olfaction generally focuses on using a sensor array with wide selectivity range, and processing methods that rely on extracting features from the sensor output, encoding them in patterns, and using pattern-matching algorithms for recognition logic [5,6].

The introduction of bioinspired neuromorphic methods for artificial olfactory systems has allowed the encoding of odor information in the form of spikes resulting in the development of computationally efficient processing methods and deployment of associated learning algorithms [3,7]. The analog VLSI circuit by Koickal et al. [8] was amongst the first silicon implementations of a neuromorphic olfactory system. Subsequently, several other contributions, such as [9,10,11,12], have implemented spike-based methods to develop neuromorphic olfactory systems. As discussed in [5], most of these studies have mainly focused on simulating the biological counterpart, thus resulting in high levels of bio-realism but poor performance parameters and impractical designs.

The bioinspired electronic nose system reported in [13,14,15,16] focuses on emulating only the underlying neuro-biological architecture that can be practically implemented in silicon rather than designing a detailed biological olfactory pathway in electronics. Furthermore, a 4 × 4 metal-oxide sensor array design implemented resistance-to-time conversion in order to generate unique rank-order signatures for classification and identification of target gases. While the use of rank-order spiking patterns was not effective for encoding information, such as changing odor concentration, they have provided a simplified platform for detecting the presence of a target gas in feature space [5,17]. However, the utilization of traditional pattern-matching methods in the presence of shuffled spike sequences imposed limitations on the performance of the classifier [17].

This paper presents an SNN-based hardware solution for continuous and rapid classification of rank-order spiking patterns in an artificial olfactory system. The key goals of this implementation are to overcome the requirement for complete rank-order frames to provide recognition information and reduce the processing latency to provide real-time classification results. The classifier was implemented using radial basis function (RBF), a subset of the nearest neighbor algorithm, on a neuromorphic pattern-matching platform where the experiments were conducted using rank-order signatures from previously implemented electronic nose systems.

## 2. Approach

While the implementation of rank-order encoding schemes has emerged as one of the more promising solutions for encoding multivariate artificial olfactory data, the performance of electronic noses that utilize this approach is hindered by the limitations of current pattern-matching methods, such as considerable computational requirements, and significant latency to provide classification results. In order to overcome these limitations, we base our approach on implementing a simplified spiking neural network (SNN) architecture that utilizes the nearest neighbor logic. This implementation has been devised to achieve the following primary objectives: (a) Continuous classification of incoming spiking patterns with minimum latency to enable real-time application. (b) Robustness to inconsistent spiking patterns resulting from shuffling of rank orders. (c) Easy integration in a rank-order-based olfactory system.

The SNN consists of 1024 leaky-integrate-and-fire (LIF) neurons that apply a bioinspired nearest neighbor approach to classify an incoming rank-order pattern. The neurons are interconnected through a daisy chain to form a network and perform classification in parallel to provide rapid recognition results. Each neuron consists of an associative memory and processing unit along with an in-built nearest neighbor-based classification logic. A crucial feature of the network is the massively parallel and bi-directional neuron bus that allows neurons in the network to collaborate and provide accurate classification results with minimum latency. The network layout and its implementation are discussed in more detail in the subsequent sections.

**Figure 1:**
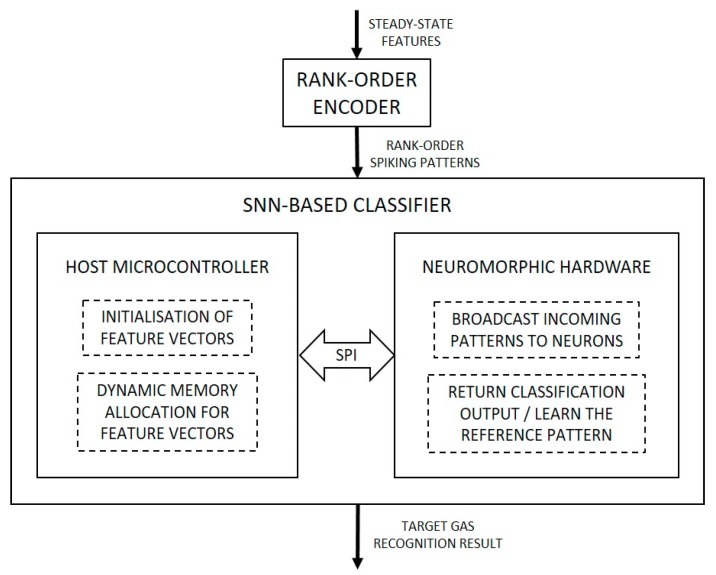
Block diagram of the classification system with its important components and sub-processes.

Since the rank-order encoding approach has been utilized only in selected artificial olfactory systems, such as [13,14,15,18,19], a limited volume of rank-order data is available for use to test the functionality of the classifier. Hence, in order to better test the robustness and quantify classifier performance, a rank-order encoder was developed using Python. The encoding logic is based on the spike latency coding described in [15] that uses sensor characteristics and power law to convert the analog output into sequences of latency spikes that represent the order in which the spikes were generated when a sensor array was exposed to a certain target gas. A subset of data points was extracted from the dataset described in [20] and used as an input for the encoder to convert the 16-array sensor output to a 16-element rank-order spiking pattern. A block diagram of the proposed approach is shown in Figure 1 below.

## 3. Rank-Order Encoding and Classifiers—Overview

The transmission of information by neurons in most early neuromorphic models was implemented using a firing rate code [21]. While the main idea of applying this approach was to include a high degree of bio-realism, the conventional rate-coding scheme was found to be incompatible with increased processing speed requirements [22,23]. As a result, rank-order spike encoding was developed as an alternative coding scheme that provided several advantages, such as speed, robustness, and ease of implementation. The rank-order spike coding represents the order in which the neurons fire in response to a stimulus, thus encoding the information in the relative timing of spikes across the neuron population. This approach was first introduced and applied by Thorpe et al. for pattern and face recognition in artificial retinas [21,23].

Building on the foundational studies presented in [13,19,24], the spike-latency coding utilized in the bioinspired microelectronic nose, as described in [14], was among the first to apply rank-order spike encoding in neuromorphic olfaction. The application of resistance-to-time conversion transforms the analog sensor responses to sequences of latency spikes. This bioinspired approach provides a unique concentration-invariant rank-order spiking pattern for a specific target gas. The rank-order signatures generated by the 4 × 4 metal-oxide sensor array for different target gases at varying concentrations is shown in [15]. The bioinspired analog gas sensing front-end, proposed by Huang and Rabaey in [18] is the most recent application of rank-order coding in artificial olfaction. A spike-timing encoding algorithm is proposed that can generate concentration-invariant temporal spike patterns, equivalent to rank-order spiking patterns, without prior knowledge of sensor characteristics. 

While the rank-order coding scheme cannot be applied to systems monitoring changes in concentration of gases, this approach is more effective in encoding multi-variate data to detect the presence of a set of target gases, especially in applications, such as bio-security and environmental monitoring [3,5]. The classifiers developed for such systems are mainly based on traditional pattern-matching techniques that compare the rank-order spiking pattern with the reference patterns stored in a library [16]. An alternative approach using conventional K-nearest neighbor (KNN) has also been proposed in [25] where a minimum spike distance algorithm was used to identify a new test rank. These techniques require an entire frame of the rank order signatures to commence the pattern-matching process, resulting in a latency build-up in result classification. Furthermore, limitations of such an approach include susceptibility to inconsistent spiking order [17], and high computational and power requirements. Recent development in a neuro-inspired spike-pattern classifier was reported in [26], but its dependency on futuristic 3D technology to apply hyper-dimensional computing principles may not be viable for current artificial olfactory systems.

## 4. Implementation Methods

### 4.1. Input Dataset

The implementation of a rank-order scheme for artificial olfactory systems has only been recently realized and remains a relatively novel concept for encoding multi-variate olfactory data. To the best of our knowledge, only selected implementations including [14,15,18] have utilized this bioinspired approach. As a result, investigations based on classification of rank-order patterns can only utilize datasets presented in these studies to validate their methodology. 

**Figure 2:**
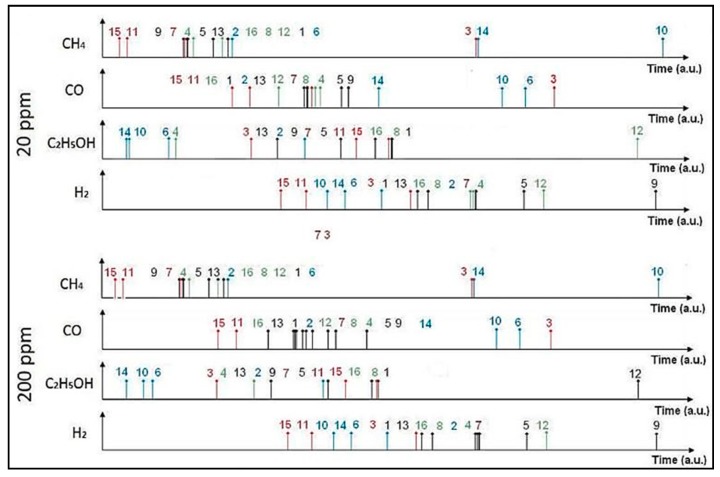
Rank-order signature for four gases, CH_4_, CO, C_2_H_5_OH, and H_2_, generated by 4 × 4 metal oxide sensor array adapted from [15].

Primary experiments to validate the classification methodology developed in this study have been conducted using the dataset, extracted from [15], consisting of eight rank-order patterns. A 16-element spike latency code (shown in Figure 2) is generated by the 4 × 4 metal-oxide sensor array using signal processing strategies that implement the resistance-to-time conversion. Rank-order patterns for four target gases, hydrogen, methane, carbon monoxide, and ethanol, at 20 ppm concentration are used as a learning dataset and spiking patterns generated at 200 ppm are used as an input dataset for validation of the methodology. Except for methane (CH_4_), the rank-order patterns for other target gases are slightly altered for the purpose of verifying the robustness of the classifier.

While this dataset met the requirements to validate classification results, the performance of the classifier could not be quantified with this limited dataset. A subset from the dataset recorded by Vergara et al. [20], was extracted from the UCI machine learning repository and encoded using rank-order spiking patterns. Along with the analog sensor output for six different analytes, several key features extracted from the output signals are included within the dataset. The normalized steady-state features extracted from analog recordings of a 4 × 4 metal-oxide gas sensor array when exposed to six different analytes namely ammonia, acetaldehyde, acetone, ethylene, ethanol, and toluene, were included in the extracted subset. The comprehensive dataset, recorded in different batches over a span of 36 months, is vast and includes data from sensor responses when exposed to a wide range of concentrations of the target gases. Furthermore, the sensor responses for target gases and concentrations are not sampled in a particular order and the distribution of the number of measurements for each target gas is inconsistent. As a result, sensor features for only a specific range of concentrations from batch 1 (Table 1) were included as part of the subset mainly because sensor characteristics change substantially over time and with exposure to higher concentrations. This results in inconsistent rank-order patterns due to several factors, such as sensor drift and aging, and substantial changes in experimental conditions. 

### 4.2. Rank-Order Encoder

The rank-order encoding methodology implemented in [13,15] was used to develop a rank-order encoder to convert the steady-state features extracted from the dataset into one-dimensional feature vectors of rank-order signatures. The steady-state features are a crucial component of this process, representing the maximal resistance change of the sensor with respect to its baseline resistance. The encoding of sensor output to spike latencies in [15] mainly focus on generating concentration-invariant rank-order patterns using sensor and gas-dependent characteristics where these properties are known in advance. Since these properties were not readily available, we computed the relative time difference between the spikes using steady-state resistance values.

The firing latency of the *i*^th^ sensor was computed using,

(1) $$t_{i}=\frac{\ln\left(R_{ij}\right)}{\gamma_{ij}}$$

where, *R_ij_* is the steady-state resistance for sensor *i* and target gas *j*, and *γ_ij_* is a constant parameter that depends on the characteristics of sensor *i* when exposed to analyte *j*. The value of the constant *γ_ij_* can either be determined using a linear regression model or by using the power law mentioned in [15]. The encoder was developed using Python and the resulting program generated an output file with rank-order codes and spike latency timings when an input file with steady-state features and the sensors’ characteristic parameters was provided.

### 4.3. Network Layout

**Figure 3:**
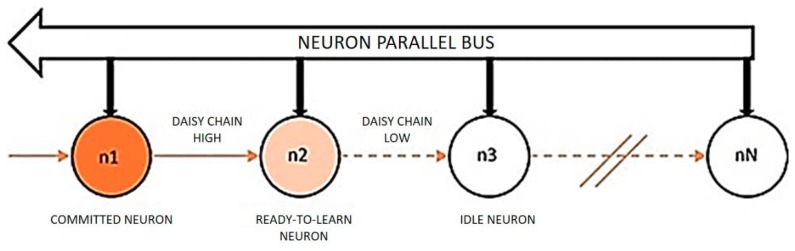
SNN layout showing neurons connected in a daisy chain.

This study utilizes the underlying SNN architecture provided by the NeuroMem chip on the BrainCard, a neuromorphic pattern-matching board [27], to implement the rank-order classifier. The classification logic is deployed on the network comprising of 1024 trainable integrate-and-fire (INF) neurons in a fully parallel daisy chain neural network as shown in Figure 3. 

**Figure 4:**
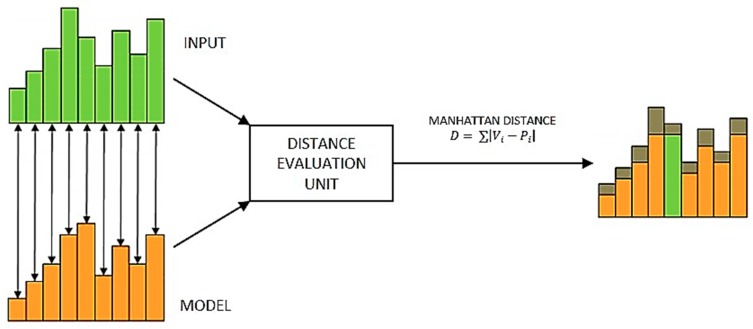
Logical implementation of distance evaluation unit based on Manhattan distance (Adapted from [28]).

The INF neurons in the network can be programmed to behave as an RBF or a KNN classifier, both subsets of the nearest neighbor logic. Along with the classification logic, each neuron has an associative memory of 256 bytes that can be used for learning rank-order feature vectors as well as for recognition of known patterns or detecting anomalies by using the learning data as a reference. A bioinspired logic is applied for classification of incoming patterns where the neurons autonomously evaluate the distance between the broadcasted and the reference pattern and generate spiking output consisting of classification information if the calculated distance is within the influence field [28]. This distance is calculated based on the Manhattan distance that evaluates each component in the patterns using the following mathematical formula:

(2) $$D=\;\sum \left|V_{i}-P_{i}\right|$$

where, $V_{i}$ is an element of the broadcasted vector and $P_{i}$ is an element of the stored reference vector. If this distance value is less than a neuron’s influence field, the neuron enters into “firing” mode and returns a positive classification with information, such as category, distance, and identifier. Figure 4 shows a model of the component by the component distance evaluation technique. The classification result is based on winner-takes-all (WTA) logic that includes recognition responses from K neurons ordered as per increasing distance values.

The neuron elements in the network are interconnected through a parallel and bidirectional neuron bus that enables the neurons to learn and process information simultaneously, and collaborate with other neurons, resulting in improved performance in terms of speed, accuracy, and flexibility. In addition to low power requirements and rapid communication over the neuron bus, this implementation benefits from the fixed processing latency (19 clock cycles) of the SNN to return the distance value of the closest neuron, which is crucial for the real-time operation of this system [28].

### 4.4. Supervised Learning

The classifier is trained using supervised learning that includes broadcasting the learning data to the neurons and setting-up their active influence field (AIF). The learning mode is activated when an input pattern is allocated a category and broadcast to the network through the neuron bus. Only the ‘firing’ neurons that have already been assigned a reference pattern and category value, and the ‘ready-to-learn’ neuron, which is the next available neuron to store the broadcasted pattern, react to the learning operation. If, for a broadcasted pattern, none of the neurons fire, a new neuron is allocated to store the reference pattern and its associated category value, and the value of its influence field is set to the current value of the maximum influence field register [28].

**Figure 5:**
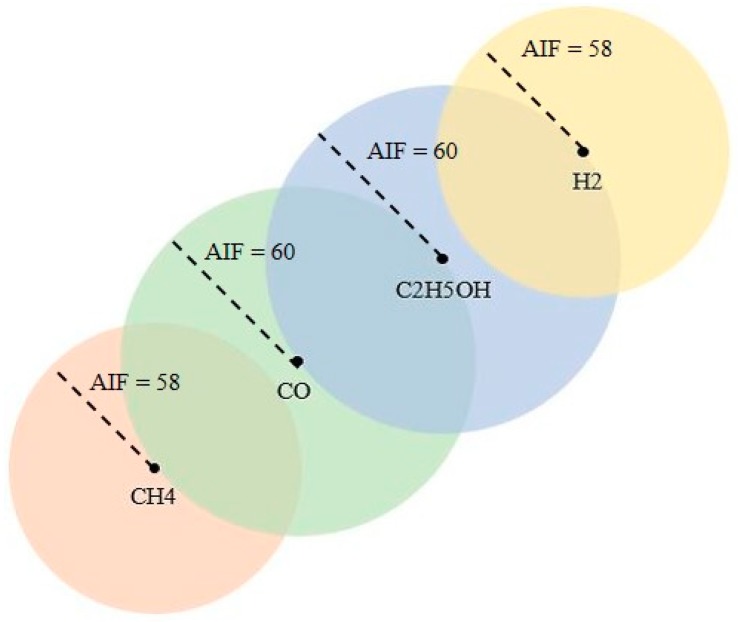
Pictorial representation of LIF neurons, with their respective active influence field (AIF), in the decision space after supervised learning of reference rank-order patterns from [15] dataset.

In the case where neurons have fired in response to a broadcasted pattern, the allocation of a new neuron to learn the reference pattern is based on the condition that none of the firing neurons should identify the broadcasted pattern as belonging to the category to learn. As a result, the influence field of this newly committed neuron is set to the distance of the closest firing neuron. Furthermore, the committed neurons initiate a corrective action to reduce their AIF value to the distance between the stored pattern and the broadcasted pattern. The feature space consisting of four committed neurons after the learning dataset from dataset [15] was broadcast is shown in Figure 5. Offline learning method was used where neurons were dynamically allocated corresponding to the number of learning patterns.

## 5. Results and Discussion

### 5.1. System Configuration

**Figure 6:**
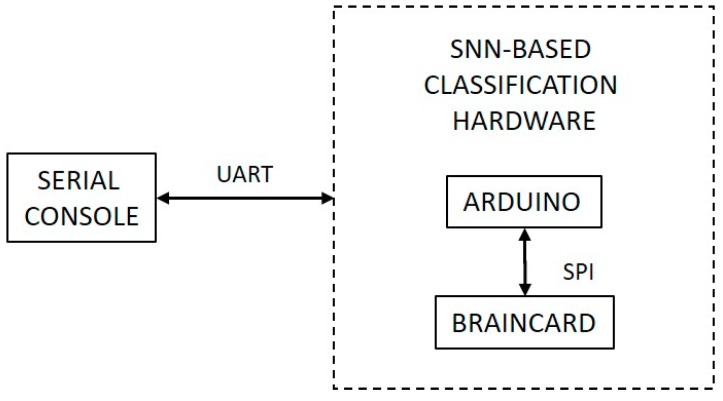
Block diagram of the hardware setup for the SNN classifier and its interfacing.

The classifier hardware setup includes interfacing of the BrainCard, neuromorphic hardware where the pattern-matching SNN is deployed, with a host microcontroller board through the serial peripheral interface (SPI) bus. The input rank-order patterns for learning and classification are transmitted from a PC-based console to the neuromorphic hardware through a serial interface provided by the host microcontroller. This emulates the implementation of the classification system as a recognition engine interfaced with a front-end sensing array that provides rank-order spiking output. A block diagram of this setup is shown in Figure 6.

Among the supported nearest neighbor algorithms, the neurons were configured for RBF recognition logic, which is more aligned to a bioinspired approach. Within this configuration, the neurons enter the ‘firing’ state only if the distance between the incoming pattern and the reference pattern is within its AIF [27,28]. This is in contrast to KNN classifiers, which always fire to input by identifying the incoming pattern to a category with minimum distance irrespective of their AIF. Such an approach leads to misclassifications and minimizes the detection of anomalies in rank-order patterns [28]. For this implementation, the value of the nearest neighbor is set to two, therefore the classification results are narrowed to two possible target gases based on the closest match of the input rank-order pattern from the rank-order signature learning data.

### 5.2. Classification Results

The experiments in this study were carried out in two stages: preliminary experiments to validate the classification methodology and experiments to quantify the performance parameters of the classifier. The preliminary experiments were conducted using the dataset extracted from [15] where rank-order signatures generated at 20 ppm for four analytes were used for learning and signatures recorded for 200 ppm were used as an input for classification. The accuracy of the classifier recorded for this dataset was 100%, and a positive recognition was obtained following the reception of 20.3% (3.25 spikes) of the total 16-element pattern frame.

Although the dataset was sufficient to demonstrate the SNN-based classification method, the performance and the robustness of the classifier was further validated using the dataset extracted from [20]. As the rank-order encoding process utilized steady-state resistance values rather than the concentration-invariant method described in [15,18], certain inconsistencies in the rank-order patterns for different concentrations were observed. This enables us to verify the classifier’s robustness to shuffling of spikes. The learning data consisting of six rank-order patterns were determined using the probabilistic rank-score coding, described in [17], which calculates the probability of a sensor spiking at a specific rank in the pattern for a target gas using

(3) $$P_{i}^{j}\left(k\right)=\frac{N_{i}^{j}\left(k\right)}{N\left(k\right)}$$

where $P_{i}^{j}\left(k\right)$ is the probability of sensor *i* spiking at rank *j* for a target gas *k*, *N*(*k*) is the total number of rank-order patterns generated for target gas *k*, and $N_{i}^{j}\left(k\right)$ is the number of times the sensor *i* in the array has spiked at rank *j* when exposed to target gas *k*. As a result, the unique learning set consisted of a reference rank-order signature for each target gas derived from this method. This learning dataset is different from the dataset used for testing the model.

**Figure 7:**
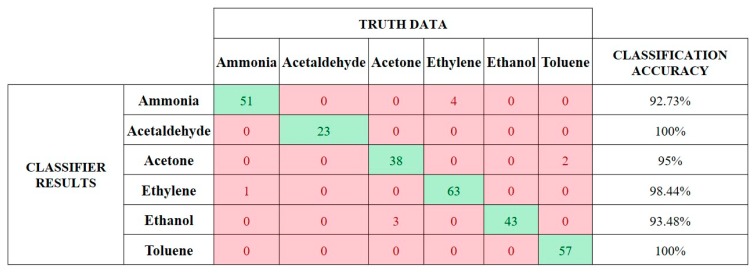
Confusion matrix showing classifier accuracy for [20] dataset.

**Figure 8:**
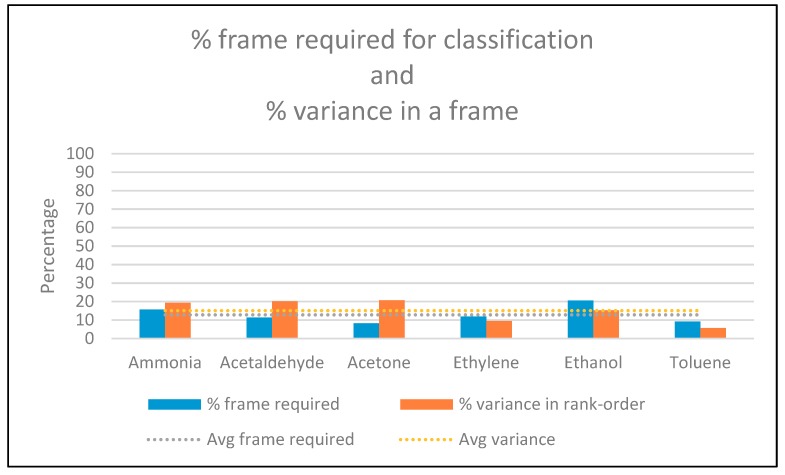
Graph plot to analyze the dependency of pattern-frame required for classification on inconsistencies in rank-order patterns observed for experiments conducted with [20] dataset.

The accuracy of the SNN classifier for 285 testing sequences of rank-order patterns for six analytes was found to be 96.5%. A confusion matrix of the classification results is shown in Figure 7. On average, the continuous classification result was obtained with the reception of 12.82% (2.05 spikes) of the total 16-element rank-order pattern frame. The average percentage of rank-order pattern frame required for each analyte along with average variance observed in each analyte is shown in Figure 8. It can be observed that the continuous classification results are not only affected by the inconsistencies in the rank-order patterns but also vary due to closely related odors that have similar elements in their rank-order signatures. For example, the similarities between rank-order signatures for ethylene and ethanol result in a requirement of 18.75% (three spikes) of the rank-order pattern frame to distinguish between the two analytes.

One of the key attributes of the SNN is its fixed processing latency of 19 clock cycles to obtain a response [28]. Hence, in both cases, the maximum latency required by the classifier to provide recognition results was observed to be 16 ms. These attributes, along with the ability of the system to continuously identify possible target gases with minimum processing latency, are crucial for an olfactory system when implemented for sensitive applications, such as biosecurity and defense.

### 5.3. Discussion

We tested the SNN classifier using two different datasets: the first dataset consisted of rank-order patterns generated as an output by an artificial olfactory system, and the second dataset was generated by encoding a subset extracted from [20] into rank-order spikes. The classification accuracy obtained for these datasets was 100% and 96.5%, respectively. Although the performance of gas identification algorithms cannot be generalized due to several factors that affect its performance, such as encoding method, selection of features, and sensing technology, based on the comparative study provided in [17], it can be observed that the proposed classifier provides higher accuracy than the 1-D rank-order classifier that recorded 55.45% with reduced power consumption and computational requirements.

The SNN-based classifier proposed in this study was able to provide continuous recognition results with each incoming rank-order spike and at a maximum processing latency of 16 ms. One of the highlights of this study is the implementation of an ‘increasing pattern-matching window’ that enables the classifier to provide recognition results as soon as the first element of the rank-order pattern is received. When an incoming spike is received by the network, the dimension of the ‘pattern-matching window’ is increased, which enables the neurons in the SNN to simultaneously compare the current rank-order spikes in the received pattern with the reference learning data. The application of this technique enables the SNN to continuously evaluate relative distances and determine the closest matching class for the broadcast pattern, with the accuracy of the decision improving as the number of received spikes increases progressively over time.

The SNN-based approach relies on the property of neurons to generate an AIF based on the learning data that is sufficient to distinguish and perform classification of incoming patterns. Based on the results obtained through these experiments, we can infer that the classifier can provide highly accurate results even when a larger input dataset with even more target gases is used, provided that the rank-order patterns for the target gases are not similar. Moreover, the use of probabilistic rank-score coding largely simplifies the process of determining the learning sequences and hence minimizes the computational overhead of traditional machine learning methods that mostly require specific and large proportions of training datasets to deliver accurate results on the testing dataset. The training dataset derived using this method consisted of a reference rank-order signature for each target gas, and, it should be noted, is completely distinct from the dataset used for testing the model. 

Along with improved accuracy with minimum computational and power requirements, the application of the SNN-based classifier enables detection of anomalies when a certain rank-order pattern is unidentified [28]. If a broadcasted pattern is not within the AIF of the committed neurons, the rank-order signature cannot be matched to a class of the reference target gases. In this case, the SNN can be programmed to store and later retrieve the rank-order signature with the ‘unknown’ category to be labeled. This approach can be particularly effective to detect consistent changes in rank-order pattern caused by factors, such as varying environmental conditions and long-term drift. 

## 6. Conclusions

Rank-order-based coding schemes have emerged as one of the more promising solutions to encode multi-variate data generated by artificial olfactory systems into temporal spiking patterns. Recent studies in this field have focused more on the encoding process, meaning the classification of the generated rank-order patterns has largely depended on statistical pattern-matching methods [25]. The performance of rank-order-based artificial olfactory systems using traditional classification methods is, however, adversely affected due to factors, such as substantial latency in providing recognition results [5].

In this paper, we report the development of SNN-based classification hardware that can be implemented as a recognition engine for rank-order-based artificial olfactory systems. The proposed technique exploits the high-speed and robust recognition capabilities of the SNN to overcome limitations of standard approaches, such as increased latency due to the requirement for a complete pattern frame to commence classification, and the reduced accuracy in cases when spikes are shuffled. The classification logic is deployed on a fully parallel silicon neural network that provides several features, such as scalability, accuracy, fixed processing latency, and low-power requirements, which are crucial enablers for integrating artificial intelligence in embedded systems.

Experiments were conducted in two stages where the classification logic was first validated using a smaller dataset extracted from an implementation of a rank-order-based artificial olfactory system [15]. The second stage of the experiment included the development of an encoder to convert the steady-state features extracted from the [20] dataset into rank-order patterns in order to evaluate the performance of the classifier. The results show that the classifier required on average only 12.82% of the total pattern frame to provide greater than 96.5% accuracy and was robust to shuffling of spiking order. The classification process of broadcasting the rank-order patterns to the neurons and returning the category of the closest firing neurons was performed with a very low processing latency of 16ms. Moreover, the classifier is able to handle anomalies and recurring unidentified temporal patterns that can be crucial to detect the effects of drift on the rank-order signatures and implement corrective logic for classification.

Current research in bioinspired olfactory systems, especially the developments in novel sensing front-ends, has provided a promising platform for the implementation of robust real-time olfactory systems [29]. Additionally, the application of spike-based neuromorphic methods for artificial olfactory systems has played a crucial role in simplifying the processing of multivariate data with low power and computational requirements. Research in neuromorphic olfactory systems is now more focused on solving issues related to portability of these systems and handling real-world data to provide recognition of target gases in real-time. The advantages of the SNN-based hardware classifier presented in this study, including continuous recognition results with minimum processing latency, and a simplified interface for using the hardware via rapid prototyping microcontroller boards, are in-line with the development of a robust electronic nose system for real-world applications.

The implementation of this hardware-based classifier, along with a sensing front-end, such as [18], that generates rank-order output, will provide a promising platform for the development of an IoT-based olfactory system, such as [30], that is portable, low power, robust, and operates in real-time. This methodology can also be extended to implement a rank-order-based neuromorphic gustatory system that can be applied to detect the presence of certain chemical components in a liquid and provide a platform for sensor fusion that includes both electronic nose and electronic tongue, as described in [31]. Future developments for this system will focus on the inclusion of the time domain for learning and recognition, analyzing the system performance in a real-world environment, and investigating acceptable limits for variance in a spiking pattern to avoid false positives during classification.

## Figures and Tables

**Table 1:** Dataset details with analytes, their concentrations and number of samples.

Analytes	Concentrations (ppmv)	Samples
Ammonia	50, 75, 100, 125, 150, 175, 200, 225, 250, 275	55
Acetaldehyde	50, 75, 100, 125, 150, 175, 200, 225, 250, 275, 300	23
Acetone	150, 200, 250, 300, 350, 400, 450, 500	40
Ethylene	50, 75, 100, 125, 150, 175, 200, 225, 250, 275	64
Ethanol	50, 75, 100, 125, 150, 175, 200, 225, 250, 275, 300	46
Toluene	20, 25, 30, 35, 40, 45, 50, 55, 60, 65, 70, 75	57
